# Experiential Avoidance as a Transdiagnostic Mediator in the Relationship Between Intolerance of Uncertainty, Maladaptive Perfectionism, and Psychiatric Symptoms: Structural and Causal Mediation Analyses in a Clinical Sample

**DOI:** 10.1002/cpp.70102

**Published:** 2025-06-19

**Authors:** Gizem Gerdan, Ebru Salcioglu

**Affiliations:** ^1^ Department of Psychology, Division of Clinical Psychology Izmir Democracy University Izmir Turkey; ^2^ Department of Psychology Istinye University Istanbul Turkey; ^3^ Department of Developmental Psychology Åbo Akademi University Vaasa Finland

**Keywords:** anxiety and depression‐related disorders, clinical sample, experiential avoidance, intolerance of uncertainty, maladaptive perfectionism, transdiagnostic factors

## Abstract

Anxiety‐ and depression‐related disorders rank among the most prevalent psychological conditions globally. Intolerance of uncertainty (IU), perfectionism, and experiential avoidance (EA) are recognized as transdiagnostic factors contributing to the development and maintenance of these emotional disturbances. However, their interplay remains insufficiently understood and has not been examined within a single research design across clinical samples with varying psychiatric diagnoses. Investigating the relationships and clinical significance of these key transdiagnostic processes in the maintenance of psychiatric symptoms represents a research priority. This study examined the mediating role of EA in the relationship between IU, maladaptive perfectionism (MP), and psychiatric symptoms (PS) using structural equation modeling (SEM) and causal mediation analyses. The sample included 221 participants (18–65 years) with a range of anxiety‐ and depression‐related diagnoses. SEM results showed that EA fully mediated the relationships between IU, MP, and PS. Increased IU and MP were linked to heightened EA, which, in turn, was associated with greater symptom severity. The model explained 54.5% of the variance in EA and 62.5% in PS. Causal mediation analyses showed that EA fully mediated the relationships between IU and PS and MP and PS in two separate models. EA explained 1.254 standard deviations in the IU‐PS relationship and 0.871 standard deviations in the MP‐PS relationship. Sensitivity analyses (*ρ* = −0.1) supported the robustness of these effects against potential confounders. These findings underscore the potential utility of assessments and interventions targeting EA to more effectively address the relationship between MP, IU, and anxiety‐ and depression‐related disorders.

**Summary:**

- This study is among the first to examine the role of EA in the relationship between intolerance of uncertainty (IU), MP, and PS in individuals with anxiety‐ and depression‐related disorders.
- IU and MP are associated with psychiatric PS severity through EA.
- Causal mediation analyses confirmed EA as a key mechanism linking IU and MP to PS, with results remaining robust after controlling for potential confounders.
- Avoidance behaviors appear to contribute to the maintenance and reinforcement of MP traits and IU, as their direct associations with PS were fully mediated by EA, resulting in increased symptom burden.
- In individuals with anxiety‐ and depression‐related disorders, assessing and addressing avoidance strategies may help reduce symptom burden in those with high IU and MP tendencies.

## 1 Introduction

Anxiety and depression are among the most common mental disorders worldwide (World Health Organization [WHO] 2022). Over 322 million people globally suffer from depression, while 264 million are affected by anxiety disorders (WHO 2017). The comorbidity of depression and anxiety has been reported to reach up to 60% (Calkins et al. 2015), with 50% of individuals with comorbidity having two disorders and the other 50% having three or more depressive and/or anxiety disorders (Ter Meulen et al. 2021). The global prevalence or burden of these disorders have not reduced since 1990; rather, a tendency toward an increase has been observed (Liu et al. 2020; Yang et al. 2021). Anxiety and depression are also among the leading causes of various disabilities and significant healthcare expenditures (James et al. 2018; Sporinova et al. 2019).

Understanding the common shared processes and mechanisms responsible for the development and maintenance of anxiety and depressive disorders is critical for developing effective treatments. The transdiagnostic approach, beyond disorder‐specific models, aims to address comorbid conditions with a single intervention and overcome the challenges of implementing multiple treatment protocols for each disorder by focusing on the shared underlying core processes of mental disorders (Mansell et al. 2009). Experiential avoidance (EA) (Fernández‐Rodríguez et al. 2018; Harvey et al. 2004), intolerance of uncertainty (IU) (Carleton 2016), and perfectionism (Egan et al. 2011) have been identified as key transdiagnostic processes implicated in the etiology, maintenance, or treatment of various psychological problems.

EA refers to efforts to escape, suppress, or modify aversive internal experiences, including negative emotions, distressing thoughts, unpleasant memories, and physiological responses (Hayes et al. 1996; Hayes et al. 2004), while also aiming to evade feared or undesirable consequences (Hofmann and Hay 2018). Although avoidance may provide temporary relief, it paradoxically increases distress over time (Bardeen 2015). As avoidance is negatively reinforced, it becomes habitual, impairs functionality, and leads to an inflexible pathological structure (Kashdan et al. 2006). The inability to stay in contact with distressing internal experiences is a key predisposition for various psychological outcomes (Bardeen 2015; Hayes et al. 1996). Both correlational (Fernández‐Rodríguez et al. 2018; Gámez et al. 2011) and experimental (De Kleine et al. 2023; Eustis et al. 2020) evidence support the link between EA and emotional disturbances. Moreover, EA is a core feature in various psychological conditions, including anxiety disorders, depression (Fernández‐Rodríguez et al. 2018; Hayes et al. 2004), obsessive‐compulsive disorder (OCD) (Den Ouden et al. 2020), post‐traumatic stress disorder (PTSD) (Hayes et al. 2004), eating disorders, and substance use disorders (Kingston et al. 2010).

IU is a cognitive predisposition characterized by negative beliefs and reactions toward uncertainty independent of the probability of its occurrence (Carleton 2012). Individuals with high IU perceive uncertainty as unacceptable and threatening, believe that uncertainty should be avoided, and experience difficulty functioning when faced with uncertainty (Buhr and Dugas 2002; Carleton 2016). The perceived absence of sufficient or key information triggers IU, increasing negative emotional states and prompting them to engage in behavioral and mental efforts to reduce uncertainty (Carleton 2012; Flores et al. 2018). Evidence has linked IU to a diverse range of psychological disorders, including anxiety disorders, depressive disorder (Hunt et al. 2022; McEvoy and Mahoney 2011), OCD, and PTSD (Hunt et al. 2022) and high IU has been reported in clinical groups (Carleton et al. 2012). Evidence shows that IU is also closely related to EA (Eisenhart‐Rothe 2022; Flores et al. 2018). High IU is stated to predispose individuals to excessive use of avoidance behaviors to make uncertain situations more predictable and enhance perception of control (Flores et al. 2018).

Maladaptive perfectionism (MP), also referred to as clinical perfectionism (Shafran et al. 2002) or negative perfectionism (Flett and Hewitt 2006), is a cognitive personality trait characterized by unrealistically high standards and excessive self‐criticism (Frost et al. 1990; Shafran et al. 2002). It is motivated by a desire to avoid failure and disappointment (Slade and Owens 1998). Individuals with MP compulsively strive to meet standards, worry about mistakes, doubt their actions, and attempt to maintain control while avoiding painful self‐criticism and distressing emotions (Moroz and Dunkley 2019; Santanello and Gardner 2007; Slade and Owens 1998). Evidence shows that MP is related to various psychiatric conditions, including anxiety disorders, depression, eating disorders, and OCD (Egan et al. 2011). Although perfectionism is considered an independent process underlying psychopathology, its clinical manifestations are closely related to EA. MP characteristics associated with psychological problems, such as doubts about actions, excessive striving for completeness, and the use of precautions and rituals to enhance perceived control, may reflect avoidance‐oriented strategies (Frost et al. 1990; Shafran et al. 2002). Studies have shown that higher MP is linked to increased EA (Moroz and Dunkley 2019; van der Kaap‐Deeder et al. 2016).

Close association of EA with IU and MP led researchers to examine EA as a mediator between these transdiagnostic processes and psychological problems. EA or avoidant coping has been shown to mediate the relationship between MP and depressive symptoms or depression (Dunkley et al. 2000, 2017; Moroz and Dunkley 2015, 2019). EA has also been identified as a mediator between MP and a range of outcomes, including pathological worry (Santanello and Gardner 2007), anxiety symptoms (Dunkley et al. 2000; Moroz and Dunkley 2019), and psychological symptoms (Dunkley and Blankstein 2000; Richard and Dunkley 2024). Furthermore, EA or avoidant coping has been found to mediate the relationship between IU and depression‐anxiety (Eisenhart‐Rothe 2022), as well as IU and metacognitive beliefs, emotional schemas, and anxiety (Akbari and Khanipour 2018), and IU and psychological symptoms (Rettie and Daniels 2021). Additionally, rumination—conceptualized as a form of cognitive avoidance (Spinhoven et al. 2016)‐ has been shown to mediate the relationship between IU and depression (Liao and Wei 2011; Huang et al. 2019; Yook et al. 2010), IU and state anxiety (Fu et al. 2023), and IU and psychological well‐being (Satici et al. 2020).

**FIGURE 1:**
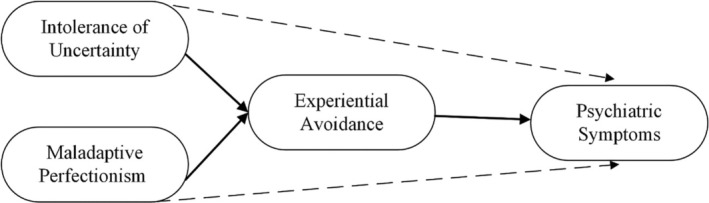
Hypothesized structural model relating intolerance of uncertainty, maladaptive perfectionism, experiential avoidance, and psychiatric symptoms.

The aforementioned studies were primarily conducted with non‐clinical samples. Studies involving clinical samples either focused solely on depression with relatively small sample sizes (*n* = 63, 71) (Dunkley et al. 2017; Yook et al. 2010) or included individuals who exhibited symptoms of anxiety and depression without a formal diagnosis (Rettie and Daniels 2021). Furthermore, no study (to our knowledge) simultaneously examined the mediating role of EA in the relationship between IU, MP, and psychiatric symptoms (PS) in clinical samples. Investigating these relationships in clinical populations is important to elucidate the relevance of the proposed transdiagnostic processes in explaining the maintenance of psychiatric symptoms. Therefore, the aim of this study was to examine the mediating role of EA in the relationships between IU, MP, and PS in individuals with various psychiatric diagnoses. We hypothesized that EA would mediate the relationship between MP, IU, and PS (see Figure 1).

## 2 Methods

### 2.1 Participants

The sample consisted of 221 participants, including 146 women (66.1%) and 75 men (33.9%), aged between 18 and 63 years (Mean Age = 33.285 ± 10.984), who were diagnosed with anxiety and depressive disorders, PTSD, or OCD and followed up at the Psychiatry Outpatient Clinic of Ondokuz Mayis University Hospital between May 2020 and April 2021. Diagnoses were made by a psychiatrist following DSM‐5 criteria. Exclusion criteria included age under 18 or over 65, illiteracy, substance abuse, personality disorders, psychotic or bipolar disorders, depression with psychotic features, and neurological conditions causing significant cognitive impairment. Two individuals with cognitive impairment confirmed by neurology consultation and five individuals who did not complete the scales or refused participation were excluded. Diagnoses within the sample included panic disorder (PD) (*n* = 30, 13.6%), generalized anxiety disorder (GAD) (*n* = 34, 15.4%), OCD (*n* = 35, 15.8%), social anxiety disorder (SAD) (*n* = 18, 8.1%), post‐traumatic stress disorder (PTSD) (*n* = 10, 4.5%), agoraphobia (*n* = 9, 4.1%), specific phobia (*n* = 9, 4.1%), illness anxiety disorder (*n* = 11, 5.0%), major depression (*n* = 48, 21.7%), comorbid anxiety and depression (*n* = 6, 2.7%), and other specified anxiety disorders (*n* = 11, 5.0%). Among participants, 41.2% (*n* = 91) had higher education (≥ 12 years), 45.2% (*n* = 100) were married, 47.1% (*n* = 104) were employed, 37.1% (*n* = 82) were using psychiatric medication (antidepressants, atypical antipsychotics, anxiolytics), and 44.3% (*n* = 98) were smokers.

### 2.2 Measures

#### 2.2.1 Multidimensional Experiential Avoidance Questionnaire‐30 (MEAQ‐30)

The original MEAQ is a 62‐item self‐report measure assessing an individual's tendency to avoid distressing negative emotional states within a multidimensional structure (Gámez et al. 2011). The shorter MEAQ‐30 (Sahdra et al. 2016) evaluates EA across six dimensions using 30 items: behavioral avoidance, distress aversion, procrastination, distraction/suppression, repression/denial, and distress endurance. Items are rated on a 7‐point Likert scale (1 = *strongly disagree*, 7 = *strongly agree*), with higher subscale scores indicating greater avoidance. The Turkish version by Ekşi et al. (2018) demonstrated internal consistency (*α* = 0.76–0.85), construct validity, and convergent validity. In this study, MEAQ‐30 subscales showed adequate internal consistency (*α* = 0.67–0.88).

#### 2.2.2 Intolerance of Uncertainty Scale‐Short Form (IUS‐12)

The IUS‐12 (Carleton et al. 2007) is a 12‐item self‐report measure that assesses individuals' reaction to uncertainty and the degree to which they find uncertainty distressing and intolerable. It comprises two subscales: prospective anxiety (7 items) and inhibitory anxiety (5 items). Items are rated on a Likert scale ranging from 1 (*not at all characteristic of me*) to 5 (*entirely characteristic of me*). The Turkish version of the scale demonstrated evidence of internal consistency (*α* = 0.88), test–retest reliability, convergent validity, and factorial stability (Sarıçam et al. 2014). The IUS‐12 showed good internal consistency in the current study (*α* = 0.89).

#### 2.2.3 Almost Perfect Scale‐Revised (APS‐R)

The APS‐R (Slaney et al. 2001) is a 23‐item self‐report measure assessing adaptive and MP through three subscales: standards, order, and discrepancy, rated on a 7‐point Likert scale (1 = *strongly disagree*, 7 = *strongly agree*). In its Turkish adaptation (Sapmaz 2006), four factors were identified: high standards, order, dissatisfaction, and discrepancy. Discrepancy and dissatisfaction represent MP, while high standards and order reflect adaptive perfectionism. Higher subscale scores indicate stronger perfectionist traits. The Turkish APS‐R demonstrated adequate construct and convergent validity, as well as internal consistency (*α* = 0.72–0.83). In here, MP demonstrated adequate internal consistency (*α* = 89).

#### 2.2.4 The Brief Symptom Inventory (BSI)

BSI (Derogatis 1993) is a 53‐item self‐report measure of psychological symptoms, which is a short version of the Symptom Check List‐90. BSI is rated on a 5‐point Likert scale (0–4), ranging from “not‐at‐all” to “extremely”. Higher scores indicate more symptoms and severe psychopathology. The Turkish version of the BSI has been shown to be valid and reliable through good internal consistency (*α* = 0.95–0.96), construct validity, and convergent validity (Sahin and Durak 1994). Different from the original form; Turkish version consisted of five factors: anxiety (13 items), depression (12 items), negative self (12 items), somatization (9 items), and hostility (7 items) (Sahin and Durak 1994). The BSI demonstrated adequate internal consistency in the present study (*α* = 0.95).

### 2.3 Procedure

The study meets the ethical standards of the Declaration of Helsinki and has been approved by the Non‐Interventional Clinical Research Ethics Board (Decision no: B.30.2.ODM.0.20.08/60‐237‐547). The scales within the scope of the study were administered to individuals who met the inclusion criteria and provided informed consent for voluntary participation. Each participant was briefed about the purpose of the study. Subsequently, the demographic information form was administered, followed by the application of the MEAQ‐30, IUS‐12, APS‐R, and BSI. To control the potential confounding effect of the presentation order, the order of administration of the scales was determined randomly for the first participant and then balanced by changing the order for the remaining participants.

### 2.4 Data Analytic Strategy

Statistical analyses were conducted using IBM SPSS 25.0, and the hypothesized model was tested via structural equation modeling (SEM) in LISREL 9.1. Normality was assessed using skewness, kurtosis, and histograms, which yielded values within ± 1, indicating a predominantly normal distribution. Outliers were identified through univariate analysis (|z| > 3.29) and multivariate analysis using the ROBPCA algorithm (Hubert et al. 2005), and the data of seven participants identified as outliers in both analyses were excluded.

Relationships among study variables were examined through Pearson correlation coefficients. The measurement model tested whether the observed variables effectively measured the latent constructs. Then, the indirect and direct relationships between the latent variables were tested with the structural equation model (SEM). Maximum Likelihood Estimation was used due to its robustness to sample size and distribution (Hu and Bentler 1995). The model included four latent variables (EA, IU, MP, PS) and 14 observed variables based on subscales. Model fit was assessed using Chi‐square (*χ* ^2^), the ratio of Chi‐square to degrees of freedom (*χ* ^2^/df), root‐mean‐square error of approximation (RMSEA), standardized root mean square residual (S‐RMR), goodness of fit index (GFI), and comparative fit index (CFI). Good fit is indicated by *χ* ^2^/df ≤ 2, *RMSEA* ≤ 0.05, *S‐RMR* ≤ 0.05, *GFI* ≥ 0.95, and *CFI* ≥ 0.95, while acceptable fit corresponds to 2 < *χ* ^2^/df ≤ 5, 0.05 ≤ *RMSEA* ≤ 0.10, 0.05 ≤ *S‐RMR* ≤ 0.08, 0.90 ≤ *GFI* < 0.95, and 0.90 ≤ *CFI* < 0.95 (Schreiber et al. 2006).

Finally, a causal mediation analysis and sensitivity analysis were conducted, including gender (Panayiotou et al. 2017), smoking status (Leventhal and Zvolensky 2015), and psychiatric medication use (Melaragno 2021) as covariates, using the mediation package in R (Tingley et al. 2014). This analysis aims to assess the mediation effect while accounting for the potential influence of other variables on the outcome. Specifically, the objective is to control for the effects of these confounders to ensure an unbiased estimation of the mediation model. Two main effects are estimated in the analysis: the average causal mediation effect (ACME) and the average direct effect (ADE). These values indicate the average effect of a one‐unit change in the predictor variable on the outcome variable, either through the mediator (ACME) or directly (ADE) (Tingley et al. 2014).

## 3 Results

### 3.1 Preliminary Statistics

Descriptive statistics and intercorrelations among study measures are presented in Table 1. There were significant correlations among subscales of EA scores, PS scores, IU scores, and MP scores. The distress endurance subscale of the MEAQ‐30 showed predominantly weak or nonsignificant associations with other research variables. Similarly, in the original MEAQ study (Gámez et al. 2011), the distress endurance subscale showed weak correlations with other subscales and low factor loadings in both clinical and healthy samples, suggesting a limited contribution to core EA features. It has been argued that distress endurance involves distinct dynamics and is not merely the inverse of avoidance. Gámez et al. (2011) also highlighted the need to directly assess avoidance behaviors and the negative appraisal of avoided experiences in EA measurement. In line with the current findings and existing literature, the behavioral avoidance, distress aversion, procrastination, distraction/suppression, and repression/denial subscales of the MEAQ‐30 were utilized as observed indicators of EA.

**TABLE 1:** Correlation matrix, means, and standard deviation of research variables.

Variables	$\bar{X}$±SS	1	2	3	4	5	6	7	8	9	10	11	12	13	14
1. Anxiety	20.076 ± 9.931	—													
2. Depression	21.755 ± 10.843	0.762***	—												
3. Negative self	16.122 ± 9.791	0.751***	0.787***	—											
4. Somatization	10.895 ± 6.857	0.709***	0.588***	0.520***	—										
5. Hostility	8.810 ± 5.463	0.664***	0.681***	0.676***	0.513***	—									
6. Behavioral avoidance	23.941 ± 6.111	0.507***	0.497***	0.506***	0.366***	0.400***	—								
7. Distress aversion	23.868 ± 6.345	0.555***	0.550***	0.591***	0.389***	0.429***	0.654***	—							
8. Procrastination	21.235 ± 6.036	0.551***	0.546***	0.512***	0.335***	0.423***	0.556***	0.579***	—						
9. Distraction/Suppression	22.583 ± 6.548	0.460***	0.442***	0.357***	0.414***	0.352***	0.620***	0.568***	0.413***	—					
10. Repression/Denial	19.140 ± 6.319	0.491***	0.470***	0.469***	0.310***	0.406***	0.485***	0.491***	0.507***	0.419***	—				
11. Distress endurance	22.429 ± 7.415	−0.242***	−0.158*	−0.138*	−0.189**	−0.023	−0.158*	−0.218**	−0.306***	0.012	−0.096	—			
12. Prospective anxiety	24.104 ± 5.725	0.410***	0.410***	0.460***	0.241***	0.395***	0.469***	0.448***	0.344***	0.454***	0.407***	0.054	—		
13. Inhibitory anxiety	16.665 ± 4.828	0.323***	0.347***	0.449***	0.233***	0.317***	0.434***	0.411***	0.299***	0.365***	0.337***	−0.128	0.683***	—	
14. Dissatisfaction	23.859 ± 8.040	0.321***	0.359***	0.447***	0.240***	0.254***	0.407***	0.429***	0.368***	0.244***	0.493***	−0.094	0.414***	0.418***	—
15. Discrepancy	25.221 ± 7.718	0.369***	0.413***	0.503***	0.221**	0.228**	0.452***	0.544***	0.441***	0.301***	0.445***	−0.197**	0.399***	0.453***	0.755***

* *p* < 0.05.

** *p* < 0.01.

*** *p* < 0.001.

### 3.2 Measurement Model

The fit indices for the measurement model of four latent variables (IU, MP, EA, PS), each with two or more observed variables indicated an acceptable to good fit; *χ* ^*2*^ = 168.076, *p* < 0.001, *χ* ^*2*^/df = 2.367, *S‐RMR* = 0.046, *RMSEA* = 0.078, 90% CI [0.063, 0.094], *CFI* = 0.978, *GFI* = 0.902. The modification indices suggested a better model fit by correlating the error variances of anxiety and somatization, both observed variables of the PS. This modification is theoretically justified, as somatic symptoms are common in depressive and anxiety disorders and could arise from emotional distress or heightened awareness of physical sensations (Bekhuis et al. 2015). Accordingly, the error variances of anxiety and somatization were correlated, resulting in a statistically significant improvement in model fit. The change in goodness of fit values was statistically significant; *∆χ* ^*2*^ = 30.221, *∆*df = 1, *p* < 0.001. The final measurement model had the following goodness of fit values ranging from good fit to acceptable fit; *χ* ^*2*^ = 137.855; *χ* ^*2*^/df = 1.969; *S‐RMR* = 0.043, *RMSEA* = 0.066, 90% CI [0.049, 0.082], *CFI* = 0.984, *GFI* = 0.918. The modified measurement model was fit to the data much better. All factor loadings of the measured variables on latent constructs were large and statistically significant (standardized values ranged from 0.632 to 0.916 and *p* < 0.001); indicating that each latent variable was well‐represented by the observed variables. The correlations among latent variables in the model were strong and statistically significant (*p* < 0.001; see Table 2).

**TABLE 2:** Correlations among latent variables.

	PS	EA	IU	MP
PS	—			
EA	0.786***	—		
IU	0.579***	0.648***	—	
MP	0.500***	0.660***	0.595***	—

*Note:* *N* = 221; PS = psychiatric symptoms; EA = experiential avoidance; IU: intolerance of uncertainty; MP: maladaptive perfectionism.

*** *p* < 0.001.

### 3.3 The Mediating Role of EA: Structural Equation Modeling

**FIGURE 2:**
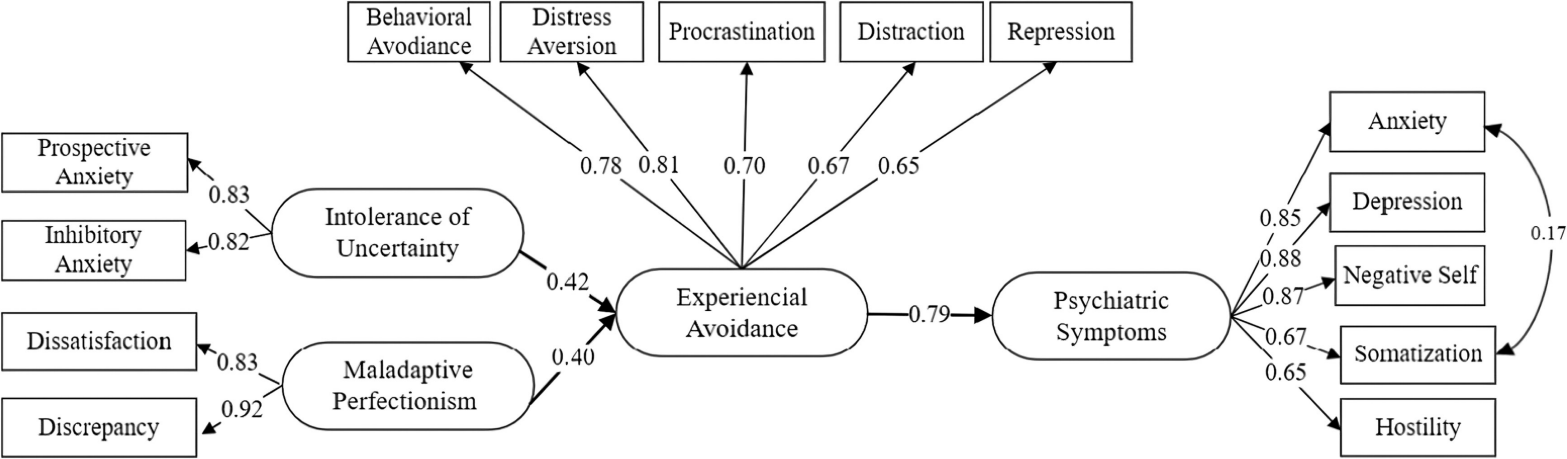
Standardized path coefficients of the final structural model. Ellipses represent latent variables. Rectangles represent observed variables. Standardized path coefficients are significant at *p* < 0.001.

The fit indices of the structural model showed either good fit (*χ* ^*2*^ = 137.855, *χ* ^*2*^/df = 1.969, *S‐RMR* = 0.043, *CFI* = 0.984) or acceptable fit (*RMSEA* = 0.066; 90% CI [0.049, 0.082], *GFI* = 0.918). *t* values showed that the paths from IU (*t* = 1.627, *p* > 0.05), and MP (*t* = −0.892, *p* > 0.05) to PS became statistically insignificant with the mediating effect of EA. While the initial estimate of the total effect of IU on PS was 0.533 (*SE* = 0.091, *p* < 0.001) and the total effect of MP on PS was 0.212 (*SE* = 0.085, *p* < 0.05), after including the mediator in the model, the direct effect of IU and MP on PS decreased to 0.141 (*SE* = 0.084, *p* > 0.05) and −0.075 (*SE* = 0.062, *p* > 0.05), respectively, both becoming statistically insignificant. After removing the insignificant paths, the *χ* ^2^ difference was found to be insignificant (*∆χ* ^*2*^ = 1.697, *∆*df = 2, *p* > 0.05), providing further evidence that these pathways do not contribute to the model after the mediating effect of EA. Goodness of fit values of the final model (Figure 2) ranged from good fit (*χ* ^*2*^ = 139.552, *χ* ^*2*^/df = 1.938, *S‐RMR* = 0.044; *CFI* = 0.984) to acceptable fit (*RMSEA* = 0.065; 90% CI [0.048, 0.081]; *GFI* = 0.917). EA fully mediated the relationship between IU, MP, and PS. The final model explained 54.5% of the variance in EA and 62.5% of the variance in PS.

### 3.4 Causal Mediation Analysis

Since causal mediation analysis can be conducted when there is one mediator, one predictor, and one outcome (Tingley et al. 2014), the mediating role of EA in the relationship between IU and PS, as well as MP and PS, was examined in two separate models (Table 3).

**TABLE 3:** Causal mediation analysis for independent variables.

	Intolerance of uncertainty			Maladaptive perfectionism		
	Estimate	95% CI^#^	*p*‐value	Estimate	95% CI^#^	*p*‐value
ACME	1.254	0.921/1.630	< 0.001	0.871	0.652/1.121	< 0.001
ADE	0.499	0.066/0.931	0.022	0.192	0.095/0.490	0.020
Total effect	1.753	1.321/2.200	< 0.001	1.064	0.754/1.372	< 0.001
Prob. Mediated	0.714	0.529/0.950	< 0.001	0.819	0.754/1.370	< 0.001
Sensitivity parameters	*ρ* = −0.1	ACME = 0		*ρ* = −0.1	ACME = 0	
	*ρ* = 0	ACME = 1.254		*ρ* = 0	ACME = 0.871	

*Note:* ACME = average causal mediation effect; ADE = average direct effect; Prob. Mediated = probability mediated; Cl# = Quasi‐Bayesian confidence interval.

EA as a mediator had significantly positive average causal mediation effects in both the relationship between IU and PS (*p* < 0.001) and MP and PS (*p* < 0.001). EA accounted for 1.254 standard deviations in the IU‐PS relationship and 0.871 standard deviations in the MP‐PS relationship. The total effects in both models (IU‐PS: *B* = 1.753, *p* < 0.001; MP‐PS: *B* = 1.064, *p* < 0.001) were fully explained by the ACME, which included all observed and unobserved mediating pathways. These findings suggest that the level of EA may account for the increased risk of PS associated with both MP and IU.

**FIGURE 3:**
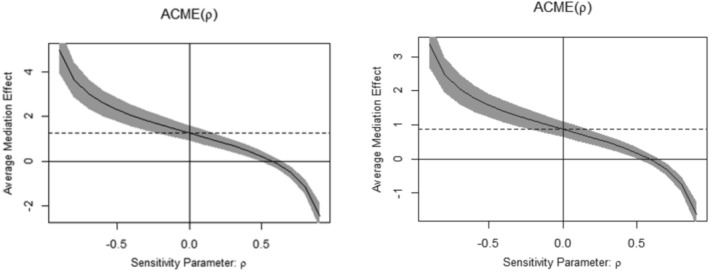
Sensitivity Analysis (left for intolerance of uncertainty, right for maladaptive perfectionism). The continuous ignorance assumption is used to calculate the ACME (*Intolerance of uncertainty* = 1.254, *Maladaptive perfectionism* = 0.871) without correlation (*ρ* = 0), as indicated by the dashed line. Keep in mind that for to become positive, *ρ* ≤ −0.1.

Moreover, assuming sequential negligibility, Figure 3 illustrates the value of the sensitivity measure *ρ* (*ρ* = −0.1) when the estimated ACME is zero. For the ACME to turn positive and show how resilient the results are to breaks in the negligibility assumption, *ρ* ≤ −0.1 is required. These results provide evidence that the model demonstrates a certain level of robustness against confounding effects.

## 4 Discussion

In this study, the mediating role of EA in the relationship between IU, MP, and PS was examined in a clinical sample. As hypothesized, higher levels of IU and MP were predictive of PS across various anxiety‐ and depressive‐related disorders. These findings are consistent with previous research demonstrating associations between IU, perfectionism, and clinical symptomatology in clinical populations (McEvoy et al. 2019; Sassaroli et al. 2008). In the current study, the relationship between MP, IU, and PS became statistically non‐significant when EA was introduced as a mediator, thus supporting the main hypothesis. The model accounted for 54.5% of the variance in EA and 62.4% of the variance in PS. Results from causal mediation and sensitivity analyses further supported the robustness of the findings. Overall, these findings suggest that as MP and IU levels increase, individuals with emotional disorders are more likely to engage in avoidance strategies, thereby contributing to the intensification of symptoms.

These findings are in line with a growing body of literature highlighting the interplay between avoidance, uncertainty, perfectionism, and psychopathology. As previously mentioned, uncertainty is a precursor to avoidance behaviors across various psychological disorders (Flores et al. 2018). It evokes significant distress in anxiety and depression, and this discomfort is closely tied to engagement in avoidance behaviors (Boswell et al. 2013; Flores et al. 2018). Individuals with anxiety‐ and depression‐related conditions often avoid situations perceived as uncertain, but also the internal experiences triggered by uncertainty, including distressing emotions, thoughts, and physiological symptoms. Avoidance strategies such as frequent checking, reassurance‐seeking, behavioral restrictions, and cognitive control via worry and rumination are employed to reduce uncertainty (Boswell et al. 2013; Carleton 2016; Fetzner et al. 2013; Flores et al. 2018; Liao and Wei 2011). In the context of psychopathology, strategies to reduce perceived uncontrollability over negative outcomes and minimize uncertainty in response to threat perception have an avoidance function. For instance, in OCD, compulsions and control behaviors are employed to enhance perceived control and certainty (Tolin et al. 2003). In GAD, excessive worry serves as a strategy to prevent or anticipate negative outcomes (Carleton 2012). In PD, behavioral avoidances and safety behaviors manage uncertainty surrounding somatic symptoms and panic attacks (Carleton et al. 2014). In SAD, avoidance and safety behaviors mitigate uncertainty about social evaluations and future judgments (Boelen and Reijntjes 2009). In PTSD, explicit or implicit avoidance strategies help cope with uncertainty about managing potential dangers (Fetzner et al. 2013). In depression, increased rumination on future negative events and depressive predictive certainty (Yook et al. 2010) similarly reflect the link between uncertainty and avoidance. While avoidance strategies may provide temporary relief, they ultimately sustain and intensify distress, contributing to symptom severity (Fernández‐Rodríguez et al. 2018) and further reinforcing IU over time. Moreover, increased avoidance behaviors also exacerbate symptom burden (Fernández‐Rodríguez et al. 2018; Hayes et al. 2004).

MP has also been closely associated with EA, particularly in the context of efforts to avoid failure, criticism, and perceived loss of control (Moroz and Dunkley 2015; van der Kaap‐Deeder et al. 2016; Richard and Dunkley 2024). Individuals with psychological conditions such as anxiety disorders, OCD (Antony et al. 1998), PTSD (Egan et al. 2014), depression (Hewitt et al. 1995), or other internalizing symptoms (Richard and Dunkley 2024), who exhibit pronounced MP traits, frequently engage in avoidance behaviors related both to their psychopathologies and perfectionistic concerns. MP characteristics—such as unrealistic expectations, doubts about actions, evaluative concerns, and fear of mistakes—often lead to disappointment, failure, self‐blame, and excessive preoccupation with perceived mistakes or shortcomings (Antony et al. 1998; Burgess and DiBartolo 2016; Shafran et al. 2002). When experiences deviate from internalized standards, negative emotions are triggered, intensifying distress, anxiety, worry, and ruminative tendencies (Burgess and DiBartolo 2016; Flett et al. 2016; Hewitt and Flett 2002). In response, individuals increasingly employ avoidance strategies to regain a sense of control and mitigate evaluative concerns, operating under the belief that mistakes or oversights could result in feared outcomes (Antony et al. 1998; Burgess and DiBartolo 2016; Flett et al. 1996). Avoidance‐focused strategies—such as repeated checking, hyper‐control, repetition, procrastination, and task abandonment—along with increased precautions, suppression of distressing emotions, and denial, are used to avoid negative experiences (Antony et al. 1998; Burgess and DiBartolo 2016; Frost et al. 1990; van der Kaap‐Deeder et al. 2016). Over time elevated emotional avoidance and increased control efforts, reinforce MP and exacerbate psychological outcomes (Burgess and DiBartolo 2016; Moroz and Dunkley 2015, 2019; Terry‐Short et al. 1995).

### 4.1 Clinical Implications

The findings of this study underscore the transdiagnostic significance of EA, demonstrating its central role in linking MP, IU, and psychopathology across a range of anxiety and depressive‐related disorders. Given the diagnostic instability observed across disorders (Hovenkamp‐Hermelink et al. 2016), the widespread prevalence and frequent comorbidity of anxiety‐depression‐related disorders (Ter Meulen et al. 2021), and the association between comorbidity and treatment‐resistance (Schaffer et al. 2012), there is a growing consensus that the focus should shift from disorder‐specific features to the identification of shared underlying core mechanisms. These findings support the adoption of a transdiagnostic approach, which may enhance both the assessment and treatment of psychological disorders by targeting core processes such as EA (Harvey et al. 2004; Mansell et al. 2009).

Building on the present findings and supporting literature, targeting EA as a core symptomatic process both in case formulation and intervention planning may enhance treatment outcomes. In assessment, focusing on the avoidance of distressing emotional experiences as a maintaining mechanism can facilitate the conceptualization of problem behaviors and associated psychological symptoms in case formulations (Salcioglu 2022). This focus on EA has important implications for intervention, as it highlights the need for therapeutic strategies that directly address avoidance tendencies. In this regard, exposure‐based therapies, which aim to reduce avoidance by promoting direct engagement with distressing internal and external stimuli, have been recognized as the gold standard for treating various psychological disorders (e.g., Hezel and Simpson 2019; Rauch et al. 2012), and their efficacy has been reported across various psychopathologies (Eustis et al. 2020). In relation to heightened IU in psychopathologies, individuals utilize cognitive and behavioral avoidance strategies to reduce uncertainty, predict outcomes, and achieve a sense of certainty. Similarly, in pathological patterns characterized by MP traits, avoidance behaviors are used to escape from negative emotions and thoughts related to high standards, unmet expectations (e.g., being flawless or comprehensive), fear of mistakes and concerns about evaluations and criticisms, and perceived control. Experimental studies and reinforcement‐learning models also provide additional causal evidence supporting the link between increased IU, MP, and increased avoidance behaviors (Aylward et al. 2019; Yiend et al. 2011). Furthermore, recent studies have demonstrated the efficacy of exposure‐based interventions specifically targeting IU (Hebert and Dugas 2019) and perfectionism (Redden et al. 2022). Therefore, in treatment approaches, it is recommended to focus on alleviating individuals' discomforting emotions, thoughts, or potential negative outcomes when examining the relationships between psychological morbidity, IU, and MP. Overall, these findings highlight the promise of transdiagnostic, exposure‐focused strategies in addressing the common avoidance‐based mechanisms that maintain psychological distress across diverse clinical representations.

### 4.2 Limitations and Future Directions

Our study has several limitations. First, its cross‐sectional design limits causal inferences. Future research should employ longitudinal or experimental designs to validate the observed relationships. Nevertheless, predictive relationships identified in the mediation analysis (Hayes 2018; Kline 2016), along with bootstrap, causal mediation, and sensitivity analyses, provide supportive evidence. These findings support the transdiagnostic nature of EA in linking IU, perfectionism, and symptom severity. Second, structured diagnostic interviews were not conducted. While this is a limitation, participants were assessed and monitored by a psychiatrist using DSM‐5‐based clinical interviews in a state hospital setting, providing reasonable diagnostic validity. Future studies incorporating structured diagnostic interviews could further enhance diagnostic rigor. Third, our sample was predominantly female. Conducting studies with a more gender‐balanced sample could enhance the generalizability of the findings. However, the influence of social gender, patriarchal norms, and heteronormative pressures on the feminization of psychopathology should not be overlooked (Roselló‐Peñaloza et al. 2019). Finally, future studies examining the relationships among MP, IU, and symptomatology—particularly the role of heightened EA strategies—would be valuable. Longitudinal designs and intervention studies targeting EA through cognitive‐behavioral therapies could not only clarify these relationships but also provide important insights into their clinical implications, contributing meaningfully to the transdiagnostic treatment literature.

## 5 Conclusion

This study is among the first to examine the relationship between IU, MP, and PS through the mediating role of EA in individuals with various anxiety‐ and depression‐related disorders. The findings demonstrate that higher levels of IU and MP are significantly associated with increased EA, and that EA, in turn, predicts greater symptom severity. These findings underscore the critical role of EA as a transdiagnostic process and highlight the potential value of assessments and interventions specifically targeting EA in addressing the relationship between MP, IU, and the symptomatology of anxiety and depression‐related disorders.

## Author Contributions

**Gizem Gerdan:** conceptualization, data curation, methodology, literature review, formal analysis, writing – original draft. **Ebru Salcioglu:** conceptualization, supervision, writing – review and editing.

## Ethics Statement

All procedures with human participants complied with institutional/national ethical standards and the 1964 Declaration of Helsinki and its amendments. The study procedures were approved by the Non‐Interventional Clinical Research Ethics Committee of Ondokuz Mayis University Hospital, the corresponding author's previous institution (Decision No: B.30.2.ODM.0.20.08/60‐237‐547).

## Consent

Informed consent was obtained from all volunteer participants involved in the study.

## Conflicts of Interest

The authors declare no conflicts of interest.

## Data Availability

The data that support the findings of this study are available from the corresponding author upon reasonable request.
